# Differential Effect of Dietary Fibers in Intestinal Health of Growing Pigs: Outcomes in the Gut Microbiota and Immune-Related Indexes

**DOI:** 10.3389/fmicb.2022.843045

**Published:** 2022-02-22

**Authors:** Yuheng Luo, Yang Liu, Hua Li, Yao Zhao, André-Denis G. Wright, Jingyi Cai, Gang Tian, Xiangbing Mao

**Affiliations:** ^1^Key Laboratory of Animal Disease-Resistant Nutrition, Ministry of Education, Sichuan Agricultural University, Chengdu, China; ^2^Key Laboratory of Animal Disease-Resistant Nutrition and Feed, Ministry of Agriculture and Rural Affairs, Sichuan Agricultural University, Chengdu, China; ^3^Key Laboratory of Animal Disease-Resistant Nutrition, Sichuan Agricultural University, Chengdu, China; ^4^Animal Nutrition Institute, Sichuan Agricultural University, Chengdu, China; ^5^University of Oklahoma, Norman, OK, United States

**Keywords:** dietary fibers, growing pig, gut microbiota, intestinal barrier, intestinal immunity

## Abstract

Although dietary fibers (DFs) have been shown to improve intestinal health in pigs, it is unclear whether this improvement varies according to the type/source of DF. In the current study, we investigated the impact of dietary supplement (15%) of pea-hull fiber (PF), oat bran (OB), and their mixture (MIX, PF, and OB each accounted for 7.5%) in the growth performance as well as intestinal barrier and immunity-related indexes in growing pigs. Twenty-four cross-bred pigs (32.42 ± 1.95 kg) were divided into four groups: CON (basal diet with no additional DF), PF, OB, and MIX. After 56 days of feeding, we found that the growth performance of PF pigs was decreased (*p* < 0.05) compared with pigs in other groups. Results of real-time polymerase chain reaction and Western blot showed that the improvement of immune-related indexes (e.g., interleukin 10 [*IL-10*]) in OB and MIX pigs mainly presented in the ileum, whereas the improvement of intestinal barrier–related indexes (e.g., *MUC1* and *MUC2*) mainly presented in the colon. Whether in the ileum or colon, such improvement of immune function may be dependent on NOD rather than TLR-associated pathways. Amplicon sequencing results showed that PF and MIX pigs shared a similar bacterial community, such as lower abundance of ileal Clostridiaceae and colonic *Streptoccocus* than that of CON pigs (*p* < 0.05). Our results indicate that OB and MIX, rather than PF, benefit the intestinal health in growing pigs, and multiple-sourced DF may reduce the adverse effect of single-soured DF on the growth performance and gut microbiota in pigs.

## Introduction

Dietary fiber (DF) was originally regarded as a component of the plant cell wall that is indigestible by human and animals (Hipsley, 1953; Trowell, 1976), and the high level of DF in the diet of pig may play an antinutritional role, such as the inhibition in the absorption of macronutrients and micronutrients (Campbell and Taverner, 1986; Zhou et al., 2013). However, recent studies show that the supplementation of some DF, such as beet pulp (Yan et al., 2017) and wheat bran (Zhao et al., 2018), may not have negative impact on the growth of animals. This might be associated with the structure of DF, which is regarded to be closely related to the intestinal development, peristalsis, homeostasis, and microbiota (Lange et al., 2010; Bach Knudsen et al., 2012).

The integrity of intestinal barrier is crucial for the maintenance of gut health. An intestinal injury rat model showed that supplementation of multiple DF reduced intestinal permeability and prevented endotoxin from entering the portal vein blood (Gao et al., 2015). Diets with high levels of high-viscosity soluble dietary fibers (SDFs), such as wheat bran and pea-hull fiber (PF), may increase the expression of mucin gene (*MUC*) and the secretion of mucin in the distal small intestine of pigs by increasing the number of goblet cells (El Kaoutari et al., 2013). However, in a rat model, the stimulation of mucin secretion by dietary supplement of low-viscosity SDF may not be related to the number of goblet cells, but was reported to be associated with an enhanced expression of *MUC* expression (Ito et al., 2009). These results indicate that DF may be important for the maintenance of mucosal barrier by regulating the number of goblet cells or the expression of *MUC*.

Although the role of DF in intestinal health has been discussed extensively, few reports focused on the comparison of different sourced DF on the intestinal barrier and immune function of animals. Most DF can be fermented by microorganisms in the hindgut of monogastric animals to produce large amounts of short-chain fatty acids (SCFAs) (El Kaoutari et al., 2013; Sonnenburg and Sonnenburg, 2014). Therefore, the effect of DF on gut health is recognized to be associated with their impact on gut microbial communities (Conlon and Bird, 2014) and the indirect physiological effects of microbial metabolites (Bach Knudsen et al., 2012).

The effects of DF on microbial composition in the gut of human or animal models have been well studied. The utilization of DF by microorganisms in human gut was considered to take place in the large intestine. However, the microbial fermentation of DF might begin early even at distal small intestine (Venema et al., 2007), which suggests that microbes in small intestine, such as ileum, should be also considered when evaluating the influence of DF on gut microbial community.

In the current study, we selected two sourced DF, PF, and oat bran (OB). The main non-starch polysaccharides (NSPs) in PF are cellulose and rhamnogalacturonan, whereas the NSPs in OB mainly are β-glucan and cellulose (Knudsen, 1997; Sajilata et al., 2006; Stephen and Phillips, 2016). In our previous study in BALB/c mice, the dietary supplement of mixed DF (half of each β-glucan and microcrystalline cellulose) had a particular impact on the colonic bacterial community and phenotype of mice compared with the sole supplement of β-glucan or microcrystalline cellulose (Luo et al., 2017). Accordingly, we attempted to investigate whether there was a similar effect of the equivalent supplement of PF and OB. Results of current study may help to further understand the underlying mechanism of different sourced DF affecting the intestinal health of pigs or other animals with similar anatomical physiology.

## Materials and Methods

All experimental procedures and animal care were performed in accordance with the Guide for the Care and Use of Laboratory Animals prepared by the Institutional Animal Care and Use Committee of Sichuan Agricultural University, and all animal protocols were approved by the Animal Care and Use Committee of Sichuan Agricultural University under permit number DKYB20131704.

### Experimental Design and Animal Feeding Management

A total of 24 cross-bred (Duroc × Landrace × Yorkshire) pigs, with an initial body weight of 32.42 ± 1.95 kg, were selected and randomly divided into four groups with six replicates in each group and one pig per replicate. Pigs in the control group (CON) were fed with basal diet without additional DF. Pigs in the other three groups were fed a basal diet with 15% PF, 15% OB, or a mixture (MIX) of OB (7.5%) and PF (7.5%), respectively. The preparation of corn-soybean meal diet was prepared according to the nutrient requirements of swine of the National Research Council (NRC) 2012 (National Research Council, 2012) and the Feeding Standard of Swine (China, NY/T65-2004) (Supplementary Table 1). The PF and OB were purchased from Shaanxi Ciyuan Biotechnology Co., Ltd. (Xi’an, China). The measured values of main nutrients including SDF and IDF of the two fibrous raw materials are shown in Supplementary Table 2. All pigs were raised in individual cages and fed three times a day (8:00 AM, 1:00 PM, and 6:00 PM). The trial lasted for 56 days, and water was available *ad libitum* during the entire experimental period.

### Reverse Transcription Real-Time Polymerase Chain Reaction Analysis of Expression of the Genes Related to Immune and Barrier Functions in the Intestinal Mucosa

At the end of the trial, all pigs were weighed before being euthanized by a lethal injection of sodium pentobarbital (200 mg/kg body weight). Digesta and mucosal samples from the middle ileum and colon of each pig were collected and stored at −80°C. Approximately 0.5 g of each mucosal sample was thawed, and total RNA was extracted using Trizol (TAKARA, Japan), and mRNA was reversely transcribed into cDNA with PrimeScript™ RT reagent kit (TAKARA, Japan). The SYBR Green–based real-time polymerase chain reaction (PCR) reaction mixture included 1.0 μL cDNA, 0.4 μL forward primer, 0.4 μL reverse primer, 5 μL SYBR Green PCR Master Mix (TAKARA, Japan), 0.2 μL correction fluid ROX (TAKARA, Japan), and 3 μL double-distilled water. Forty cycles of PCR were conducted as follows: predenaturation at 95°C for 10 s followed by 5 s of denaturation at same temperature, annealing at 60°C for 20 s, and extension at 72°C for 15 s (ABI 7900, United States). Three housekeeping genes, β-actin, 18S rRNA, and GAPDH, were selected as internal references, and relative quantity of each target gene in each sample was calculated according to the method (Larionov et al., 2005). The sequences of primers and the length of the PCR product for each gene are shown in Supplementary Table 3.

### Western Blotting Analysis of TLR4 and Nuclear Factor κB Expression in the Intestinal Mucosa

The proteins from each mucosal sample were extracted according to described method (Kim et al., 2012). Expression of targeting proteins, TLR4 and nuclear factor κB (NF-κB), was analyzed using Western blotting as previously described (Finamore et al., 2014) with β-actin as the internal reference. The bands were analyzed with Bio-Rad image analysis system, and the formula of separating and concentrated gel is shown in Supplementary Table 4.

### Analysis of the Bacterial Community and Concentrations of Short-Chain Fatty Acids in the Digesta Samples

Procedures for the extraction of genomic DNA, analysis of microbial community, and the measurement of SCFAs concentrations have been previously described (Luo et al., 2015). Briefly, pairs of reads from the original DNA fragments were first merged using FLASH (Fast Length Adjustment of SHort reads) (Magoč and Salzberg, 2011). Diversity was analyzed using the QIIME (Quantitative Insights Into Microbial Ecology) software (Caporaso et al., 2010) after removal of chimeric sequences using USEARCH (Edgar et al., 2011). Operational taxonomic units (OTUs) for the sequences were picked using the *de novo* OTU picking protocol with a 97% similarity threshold. The bacterial taxa differentially represented between groups were identified using LEfSe [linear discriminant analysis (LDA) coupled with effect size] (Segata et al., 2011). All reads were deposited in the BIG Data Center in Beijing Institute of Genomics and can be accessed in the Genome Sequence Archive under accession number CRA001323^1^. The concentration of acetate, propionate, and butyrate in the digesta samples was measured using a gas chromatograph (GC) (GC-14B, Shimadzu, Japan; capillary column: 30 m × 0.32 mm × 0.25-μm film thickness), and the minimal detectable limit for each SCFA was 0.01 mmol/L.

### Statistical Analysis

Data on the growth performance, expression of related genes and proteins, concentrations of SCFAs, and the relative abundance of main bacterial phyla and genera were first checked for normal distribution using the Descriptive Statistics (Explore) module of software SPSS 16.0 (SPSS Inc., Chicago, IL, United States). For those data with normal distribution, one-way analysis of variance was used to analyze the difference among groups, and the *post hoc* multiple-comparisons test was then performed using the Bonferroni method. The homogeneity of variance was tested using Duncan analysis, whereas Kruskal–Wallis non-parametric test was used to analyze the difference between groups for those skewed data. The correlation between the SCFA concentration and bacterial species was calculated using a Pearson correlational analysis, and the results were visualized using vegan, ggcor, and dplyr packages in R 4.0.1. Differences were considered to be significant when *p* < 0.05 and not significant when *p* ≥ 0.05.

## Results

### The Growth Performance of Pigs in Different Groups

At the beginning of the experiment, there was no significant difference in the initial weight of pigs among the four groups (*p* > 0.05, Supplementary Table 5), indicating the rationality of grouping by body weight. Compared with CON, OB, and MIX pigs, the final body weight, feed intake, and body weight gain of PF pigs were decreased (*p* < 0.05).

### The Expression of Genes Related to Intestinal Barrier and Immune Function of Pigs in Different Groups

**FIGURE 1:**
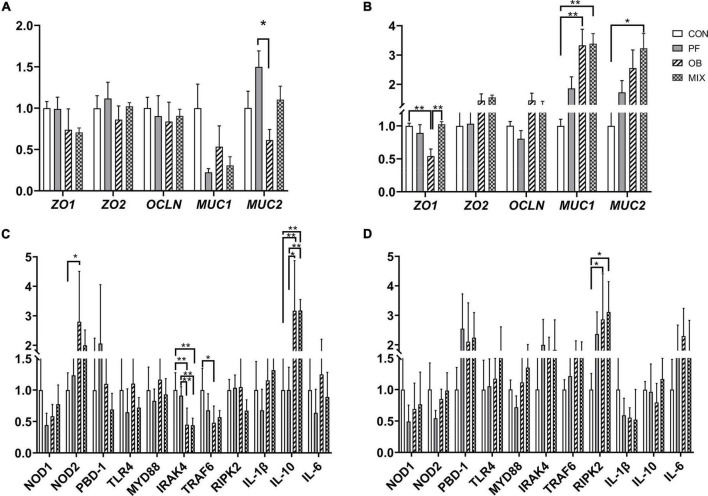
The relative expression of intestinal barrier and immunity-related genes in the ileum and colon of pigs fed different diets. **(A)** The expression of genes encoding tight junction proteins and mucin in the ileal mucosa. **(B)** The expression of genes encoding tight junction proteins and mucin in the colonic mucosa. **(C)** The expression of key genes associated with TLR4 and NOD pathways in the ileal mucosa. **(D)** The expression of key genes associated with TLR4 and NOD pathways in the colonic mucosa. *p < 0.05, **p < 0.01. CON, control group; PF, 15% pea-hull fiber in the diet; OB, 15% oat bran in the diet; MIX, 7.5% pea-hull fiber and 7.5% oat bran in the diet.

For the ileal mucosa, we did not find any difference in the expression of the five intestinal barrier–related genes between CON group and fiber supplemented group (*p* > 0.05, Figure 1A), but the expression of *MUC2* in PF pigs was higher than that in OB pigs (*p* < 0.05, Figure 1A). For the colonic mucosa, the expression of *MUC1* in PF and MIX pigs, as well as the expression of *MUC2* in MIX pigs, was increased compared with CON pigs (*p* < 0.05, Figure 1B), whereas the expression of *ZO-1* in OB pigs was lower than that in CON and MIX pigs (*p* < 0.05, Figure 1B).

For the investigated genes related to immune function, we found the expression of *IRAK4* in the ileum of OB and MIX pigs, and the expression of *TRAF6* in the ileum of OB pigs was decreased compared with CON pigs (*p* < 0.05, Figure 1C), whereas the expression of *IL-10* in the ileum of OB and MIX pigs was increased compared with CON and PF pigs (*p* < 0.05, Figure 1C). Interestingly, the expression of *NOD2* in the ileum of OB pigs was higher than that of PF pigs (*p* < 0.05, Figure 1C), but the expression of *IRAK4* in the ileum of OB and MIX pigs was lower than that of PF pigs (*p* < 0.05, Figure 1C). However, only the expression of *RIPK2* in OB and MIX pigs was found higher than that in CON pigs (*p* < 0.05, Figure 1D) in the colon.

### The Abundance of Protein TLR4 and NF-κB in the Ileum and Colon of the Pigs in Different Groups

**FIGURE 2:**
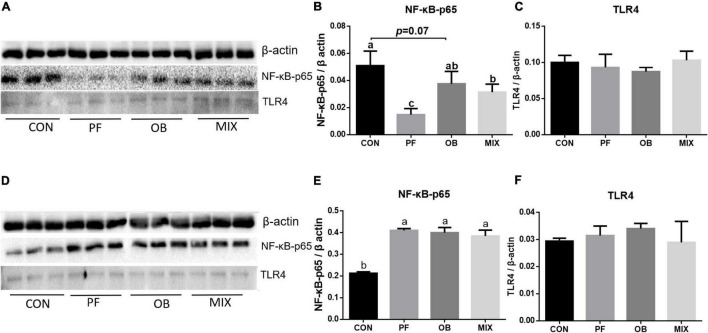
The expression of TLR4 and NF-κB proteins in the ileal and colonic tissues of growing pigs in CON, PF, OB, and MIX groups. **(A)** The profile of sodium dodecyl sulfate–polyacrylamide gel electrophoresis (SDS-PAGE) of β-actin, NF-κB–p65, and TLR4 proteins in the ileal mucosa. **(B)** The average relative abundance (fold) of protein NF-κB–p65 to β-actin in the ileal mucosa. **(C)** The average relative abundance (fold) of protein TLR4 to β-actin in the ileal mucosa. **(D)** The profile of SDS-PAGE of β-actin, NF-κB–p65, and TLR4 proteins in the colonic mucosa. **(E)** The average relative abundance (fold) of protein NF-κB–p65 to β-actin in the colonic mucosa. **(F)** The average relative abundance (fold) of protein TLR4 to β-actin in the colonic mucosa. Different lowercase letters in the bar graphs indicate significant differences between two groups (*p* < 0.05, *n* = 3). CON, control group; PF, 15% pea-hull fiber in the diet; OB, 15% oat bran in the diet; MIX, 7.5% pea-hull fiber and 7.5% oat bran in the diet.

No difference in the abundance of protein TLR4 was found in both ileum and colon of pigs among the four groups (*p* > 0.05, Figures 2A–F). Compared with CON pigs, the abundance of NF-κB–p65 in the ileum of PF and MIX pigs was decreased (*p* < 0.05, Figure 2B), whereas its abundance in the colon of PF, OB, and MIX pigs was increased (*p* < 0.05, Figure 2E). Moreover, the abundance of NF-κB–p65 protein in the ileum of OB pigs tended to be lower than that of CON pigs (*P* = 0.07, Figure 2B).

### The Concentration of Short-Chain Fatty Acids in the Ileal and Colonic Digesta of Pigs in Different Groups

**FIGURE 3:**
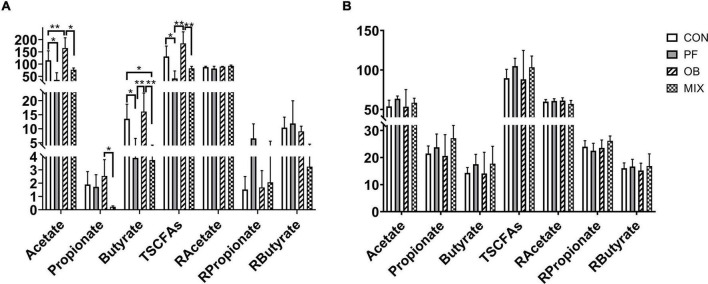
The concentration and proportion of SCFAs in the ileal and colonic digesta of pigs in different groups. **(A)** The concentration and proportion of SCFAs in the ileum of the pigs. **(B)** The concentration and proportion of SCFAs in the colon of the pigs. *p < 0.05, **p < 0.01. CON, control group; PF, 15% pea-hull fiber in the diet; OB, 15% oat bran in the diet; MIX, 7.5% pea-hull fiber and 7.5% oat bran in the diet; TSCFAs, total SCFAs; R, ratio of each SCFA to TSCFAs.

Results of GC analysis showed that changes in the concentration of SCFAs induced by DF supplement were concentrated in the ileum (Figure 3A) rather than the colon (Figure 3B). In detail, when compared with CON pigs, the concentration of acetate and total SCFAs (TSCFAs, the sum of acetate, propionate, and butyrate concentrations) in the ileum of OB pigs was increased (*p* < 0.05), whereas the concentration of acetate, butyrate, and TSCFAs in the ileum of PF pigs, as well as the concentration of butyrate in the ileum of MIX pigs, was decreased (*p* < 0.05, Figure 3A). On the contrary, no difference in the concentration of SCFAs was found in the colon of the pigs among groups (*p* > 0.05, Figure 3B). Interestingly, the ratio of each SCFA to TSCFAs showed no difference (*p* > 0.05, Figures 3A,B), whether in ileum or colon.

### The Bacterial Community in the Ileal and Colonic Digesta of Pigs in Different Groups

No significant difference was found in the Shannon index of microbial community in both ileal and colonic digesta of pigs among the four groups (*p* > 0.05, Table 1). Alternatively, the unweighted-Unifrac distance of ileal samples in PF pigs was higher than that in MIX pigs (*p* < 0.05). Compared with CON pigs, the unweighted-Unifrac distance of colonic samples in MIX pigs was higher (*p* < 0.05).

**TABLE 1:** The α- and β-diversity of microbial community in the digesta samples from ileum and colon of pigs in CON, PF, OB, and MIX groups^1^.

Item	Ileum						Colon					
	CON	PF	OB	MIX	SEM	*p*-value	CON	PF	OB	MIX	SEM	*p*-value
Shannon index	4.19	3.65	3.20	3.66	0.14	0.10	7.63	7.25	7.81	7.55	0.12	0.42
Unweighted-unifrac distance	0.650^ab^	0.661^a^	0.599^ab^	0.588^b^	0.01	0.02	0.563^b^	0.588^ab^	0.589^ab^	0.606^a^	0.01	0.03

*^1^The variant alphabetical superscript in the same row indicates significant difference between groups when p < 0.05, n = 6. CON, control group; PF, 15% pea-hull fiber in the diet; OB, 15% oat bran in the diet; MIX, 7.5% pea-hull fiber and 7.5% oat bran in the diet; SEM, standard error of mean.*

At phylum level (Supplementary Table 6 and Supplementary Figure 1), only the relative abundance of phylum WPS-2 in the colonic digesta of PF pigs was increased compared with pigs in other groups (*p* < 0.01). Beyond that, the abundance of other phyla in the ileal and colonic digesta of pigs was not different among groups (*p* > 0.05).

At genus level, we found that the abundance of *Turicibacter* in PF pigs and the abundance of an unidentified genus belonging to Clostridiaceae in the ileal digesta of PF and MIX pigs were decreased compared with CON pigs (*p* < 0.05, Supplementary Table 7). And compared with OB pigs, the abundance of an unidentified genus belonging to Clostridiaceae was decreased in PF and MIX pigs (*p* < 0.01). In the colonic digesta, the abundance of CF231 and *Streptococcus* in PF and MIX pigs showed a decrease compared with CON pigs (*p* < 0.05). In addition, the abundance of an unidentified genus belonging to Clostridiaceae was also decreased in PF pigs than that in OB pigs (*p* < 0.05).

According to the results of LDA and LEfSe analysis, a total of 12 bacterial taxa in ileal digesta and 9 bacterial taxa in colonic digesta were different in relative abundance (α = 0.01, LDA score > 3.0) in pigs among different groups. In the ileal digesta, the genus *Turicibacter* and an unknown genus of Clostridiaceae were more abundant in CON pigs than in other pigs (*p* < 0.05, Supplementary Figure 2), whereas the order Lactobacillales and genus *Candidatus Arthromitus* were more abundant in PF pigs compared with those in pigs of other groups (*p* < 0.05, Supplementary Figure 2). In the colonic digesta, the families Streptococcaceae and Paraprevotellaceae, as well as the genera *Streptococcus* and CF231, showed more enriched in CON pigs compared with other pigs (*p* < 0.05, Supplementary Figure 3). Compared with CON, OB, and MIX pigs, the genus *Lachnospira* was more abundant in PF pigs (*p* < 0.05), whereas the family Clostridiaceae and phylum WPS were enriched in OB pigs compared with other pigs (*p* < 0.05, Supplementary Figure 3).

### Correlation Between the Concentration of Short-Chain Fatty Acids and the Abundance of the Main Bacterial Groups

**FIGURE 4:**
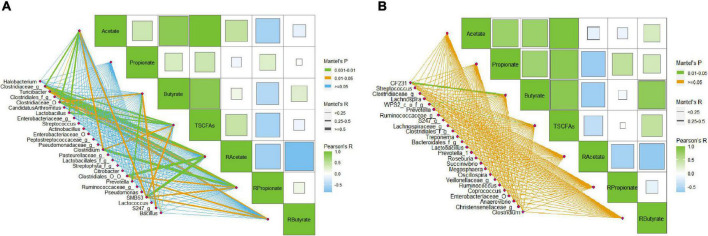
Pearson correlation between the relative abundance of main bacteria and SCFAs in the ileal and colonic digesta of the growing pigs. **(A)** Correlation between the abundance of main bacteria (top 20 bacterial genera and bacteria with significant difference between groups) and SCFA concentrations and ratios in the ileal digesta of the pigs; **(B)** correlation between the abundance of main bacteria (top 20 bacterial genera and bacteria with significant difference between groups) and SCFA concentrations and ratios in the colonic digesta of the pigs. The lowercase letters o, f, and g following the Latin names of bacteria mean order, family, and genus, respectively.

Many species of bacteria in swine gut can ferment complex carbohydrates to produce SCFAs. The results above indicate that dietary supplement of DF may alter the concentrations and ratio of SCFAs in the gut, especially in the ileum, of the growing pigs. To further clarify the relationship between the changes in SCFAs and the composition of microbiota, a Pearson correlation analysis was conducted. We found that the abundance of the main bacterial genera (top 20 and significantly different genera) in ileal digesta of the pigs showed an extensive correlation with the concentration of acetate, butyrate, and propionate, as well as the ratio of each SCFA (Figure 4). For example, six bacterial groups, including *Lactobacillus*, *Clostridium*, and SMB53, were correlated with the concentration of acetate, whereas another six genera, including *Lactobacillus*, *Prevotella*, and *Pseudomonas*, were related to the concentration of butyrate (Figure 1A). In a word, several known genera such as *Turicibacter*, *Lactobacillus*, *Clostridium*, *Prevotella*, *Bacillus*, and SMB53 presented strong correlations with the concentration or ratio of SCFAs in the ileum. Unlike that, we found only two genera that might be associated with the concentration of butyrate (Figure 4B).

## Discussion

Although DFs have been regarded beneficial to humans and animals by reducing the incidence of many diseases, such as diabetes, cardiovascular disease, colorectal cancer, and obesity (King, 2005; Papathanasopoulos and Camilleri, 2010; Xu et al., 2014), few studies have focused on the mechanisms of different-sourced DF acting on the intestinal mucosal immunity and barrier functions. The current study revealed that the body weight gain of growing pigs was reduced by the dietary supplement of PF. However, the finally measured digestible energy of PF-containing diet (3.96 MJ/kg) was even slightly higher than that of control diet (3.78 MJ/kg), indicating that the decrease in weight gain of these animals may be due to the decline of feed intake, which probably resulted from the high concentration (15%) of PF. Despite the adverse impact of PF on the weight gain of the pigs, the intestinal barrier or immunity-related indexes of the pigs were, more or less, improved by the high-level supplement of these DF. Interestingly, the effect of these DF was different, depending on the intestinal segment.

We also investigated the relative expression of five intestinal barrier–related genes. We found that in the ileum of the pigs, none of these genes were differently expressed in pigs fed DF-containing diets compared with control pigs. Alternatively, the expression of several genes involved in the mucosal immunity was changed in the intestine of these animals. The transmembrane TLR4 and NOD are two proteins important in recognizing the pathogen-associated molecular patterns to regulate the innate immune responses (Liu et al., 2013). Binding of TLR4 or NOD with ligands can ultimately activate NF-κB and in turn induce the expression of inflammatory mediators (Fukata et al., 2009), such as IL-1β, IL-6, and IL-10. Appropriate levels of IL-1β can participate in immune regulation, but its high expression may induce tissue damage (Dionne et al., 1998). IL-10 can promote differentiation and proliferation of B cells, secretion of antibodies, and inhibition of inflammatory and cellular immune responses and also can enhance the tolerance associated with adaptive immunity and immune clearance (Antosz et al., 2015). In the current study, although the mRNA level of IL-6 in all mucosal samples kept stable, the expression of *IL-10* in the ileal mucosa was remarkably increased in those pigs fed OB and mixed DF-containing diets. Further analysis showed that the expression of *NOD2*, one of the key genes on NOD-associated pathway, in the ileal mucosa of pigs fed OB-containing diet was increased. Meanwhile, the expression of *TRAF6* and *IRAK4*, two key genes on TLR4-associated pathway, in the ileum of pigs fed OB and/or mixed fiber–containing diets was decreased, which was not accompanied by changes in the abundance of proteins TLR4 and NF-κB–p65. These results indicate that the improvement of ileal immune function by OB or mixed fibers may depend on NOD2-associated signal pathway rather than TLR4-associated pathway. Both bacteria and fungi in animal gut are the ligands of TLR and NOD (Creagh and O’Neill, 2006; Wolf et al., 2011; Netea et al., 2015). Microbes in the gastrointestinal tract are the main subjects for utilization of DF. In the current study, although the Shannon index of microbial community in the ileal digesta of the pigs fed DF-containing diets was observed to be decreased without a statistical difference, the range of decline varied from 12.6 to 23.6%, especially in those pigs fed OB-containing diet. This consequently suggests that the reduction of specific microorganisms may contribute to the decreased expression of genes associated with NOD signaling pathways, which in turn resulted in the increased expression of anti-inflammatory cytokine *IL-10*. On the other hand, the decline in the diversity or even the quantity of ileal microorganisms may be beneficial to the intestinal health of host. A large number of reports have confirmed that eliminating small bowel bacterial overgrowth can alleviate some intestinal inflammation including irritable bowel syndrome (Ghoshal et al., 2017). Sequencing results showed that the abundance of certain bacterial groups, such as Clostridiaceae, the major acetate producers in the hindgut of human and monogastric animals (Rey et al., 2010; Louis et al., 2014), decreased in the ileal digesta of pigs fed PF or mixed fiber–containing diets, which might directly lead to the decrease in the absolute concentration of ileal acetate and butyrate in these pigs. However, this decline may be compensated for the increase in some other SCFA-producing bacteria, such as Lactobacillales, to keep the ratio of these SCFAs to TSCFAs invariable. Interestingly, we not only found a greater change in the concentration of SCFAs in the ileum than that in the colon of those pigs fed DF-containing diets, but also revealed a stronger correlation between such change with the abundance of specific bacterial groups, such as *Lactobacillus*, *Prevotella*, and *Clostridium*. The results indicate that these bacteria may probably be the contributors of the changes in SCFA concentration coursed by DF-containing diets. Different from that observed in the ileum, both the expression of *MUC1* and *MUC2* in the colonic mucosa of pigs fed OB and/or mixed fiber–containing diets were increased compared with control pigs, indicating an enhanced barrier function in the colon. In addition, the expression of NF-κB protein, but not TLR, in the colonic mucosa of pigs fed DF-containing diets was markedly increased than control pigs. Our further analysis showed that the expression of *RIPK2* on the NOD-associated pathway increased in the colon of pigs fed diets with OB or mixed DF, suggesting that the increased expression of NF-κB protein may be mediated by the genes involved in NOD signaling pathway in the colon. Accordingly, both the α-diversity and β-diversity of the microbial community in the colonic digesta of pigs fed DF-containing diets were increased. It is worth mentioning the abundance of genus *Lachnospira* in the colon of pigs fed PF and mixed fiber–containing diets, accompanied by the decrease in *Streptococcus*, whereas the supplement of OB in the diet specifically increased the abundance of Clostridiaceae. It is reported that genus *Lachnospira* exists widely in the gastrointestinal tract and can utilize some DF such as pectin (Dusková and Marounek, 2001). Bacteria categorized as family *Lachnospiraceae*, including *Lachnospira*, *Butyrivibrio*, and *Roseburia*, are generally regarded as beneficial microorganisms producing butyrate and propionate (Cotta and Forster, 2006). In this respect, PF-containing diet may promote the proliferation of potential probiotics and inhibit the growth of specific conditional pathogens in the colon of pigs, which may have a positive effect on the intestinal health of these animals.

In summary, our results showed that the dietary supplement (15%) of OB or the equivalent mixtures of PF and OB, rather than the sole supplement of PF, may benefit the gut health in growing pigs. However, such positive effects may vary, depending on the intestinal segment; that is, the immune-related indexes are mainly improved in the ileum, whereas the intestinal barrier–related indexes are mainly improved in the colon. What needs to be pointed out is that the improvement of immune function in these pigs seems to be dependent on NOD rather than TLR-associated pathways, whether in the ileum or colon. Although the polysaccharides from DF can only be degraded by microorganisms in gastrointestinal tract, the supplement of these DF had limited effects on the microbial diversity in the ileum and colon of experimental pigs. Despite that, bacterial groups that specifically responded to each DF in the ileal or colonic digesta were identified. Contrary to general cognition, the impact of these DF on the concentration of SCFAs in the ileal digesta of the pigs was greater than that in the colon. Although the absolute concentration of acetate, propionate, and butyrate was decreased in the ileum of pigs fed diets with DF, the proportion of these SCFAs was not changed. In addition, a direct correlation between the change of microbial community and the expression of genes associated with intestinal barrier and immunity was not found. In terms of microbial community and composition, PF and mixed DF rather than OB showed similar effects.

## Data Availability Statement

The datasets presented in this study can be found in online repositories. The names of the repository/repositories and accession number(s) can be found below: https://ngdc.cncb.ac.cn/gsa/browse/CRA001323, CRA001323.

## Ethics Statement

The animal study was reviewed and approved by all experimental procedures and animal care were performed in accordance with the Guide for the Care and Use of Laboratory Animals prepared by the Institutional Animal Care and Use Committee of Sichuan Agricultural University, and all animal protocols were approved by the Animal Care and Use Committee of Sichuan Agricultural University under permit number DKYB20131704.

## Author Contributions

YhL designed the experiments, wrote the manuscript, and had primary responsibility for the final content. YL helped to write the manuscript and analyzed part of the data. HL conducted the data analysis of sequencing and flow cytometry. YZ conducted the animal trial, real-time PCR analysis, and Western blot. A-DW helped to collect references and revised the manuscript. JC, GT, and XM helped to analyze the data. All authors contributed to the article and approved the submitted version.

## Conflict of Interest

The authors declare that the research was conducted in the absence of any commercial or financial relationships that could be construed as a potential conflict of interest.

## Publisher’s Note

All claims expressed in this article are solely those of the authors and do not necessarily represent those of their affiliated organizations, or those of the publisher, the editors and the reviewers. Any product that may be evaluated in this article, or claim that may be made by its manufacturer, is not guaranteed or endorsed by the publisher.

## Footnotes

1
https://ngdc.cncb.ac.cn/gsa/browse/CRA001323
