# Supplementary material for: Differential Effect of Dietary Fibers in Intestinal Health of Growing Pigs: Outcomes in the Gut Microbiota and Immune-Related Indexes
Source: Front Microbiol. 2022 Feb 22;13:843045. doi: 10.3389/fmicb.2022.843045 (PMC8902361; doi:10.3389/fmicb.2022.843045)
Supplement: Supplementary file 1 [file Data_Sheet_1.doc]

**Supplemental Table 1** The composition and nutritional value of experimental diets (air-dry basis)

| Ingredients (g/kg diet) | CON | PF | OB | MIX |
| --- | --- | --- | --- | --- |
| Corn | 520 | 351 | 351 | 351 |
| Soybean meal | 200 | 200 | 200 | 200 |
| Soybean oil | 3.00 | 35.0 | 35.0 | 35.0 |
| Corn starch | 200 | 200 | 200 | 200 |
| Pea fiber | - | 150 | - | 75.0 |
| Oat bran | - | - | 150 | 75.0 |
| Sugar | 20.0 | 20.0 | 20.0 | 20.0 |
| Soy protein concentrate | 24.0 | 10.0 | 10.0 | 10.0 |
| L-Lysine | 3.90 | 4.30 | 4.30 | 4.30 |
| DL-Methionine | 1.40 | 1.70 | 1.70 | 1.70 |
| L-Threonine | 2.00 | 2.00 | 2.00 | 2.00 |
| Limestone | 12.0 | 12.0 | 12.0 | 12.0 |
| CaHPO_4_ | 7.50 | 7.50 | 7.50 | 7.50 |
| NaCl | 3.00 | 3.00 | 3.00 | 3.00 |
| Choline chloride | 1.00 | 1.00 | 1.00 | 1.00 |
| Mineral premix^1^ | 2.00 | 2.00 | 2.00 | 2.00 |
| Vitamin^2^ | 0.300 | 0.300 | 0.300 | 0.300 |
| Total (kg) | 1.00 | 1.00 | 1.00 | 1.00 |
| Nutritional value^3^ | | | | |
| Digestive energy (DE), MJ/kg | 3.78 | 3.96 | 4.08 | 4.01 |
| Crude protein (CP) | 133 | 116 | 139 | 131 |
| Ether extract (EE) | 25.7 | 50.0 | 57.6 | 59.4 |
| Crude fiber (CF) | 25.4 | 98.6 | 32.1 | 64.3 |
| Neutral detergent fibers (NDF) | 294 | 286 | 248 | 341 |
| Acid detergent fiber (ADF) | 46.0 | 112 | 80.7 | 105 |
| Soluble dietary fiber (SDF) | 21.1 | 30.5 | 48.3 | 41.7 |
| Insoluble dietary fiber (IDF) | 173 | 259 | 215 | 253 |
| Total dietary fiber (DF)^4^ | 194 | 290 | 263 | 295 |
| Calcium | 7.00 | 7.10 | 7.10 | 7.10 |
| Available phosphorus (AP) | 2.10 | 2.20 | 2.20 | 2.20 |
| Lysine | 9.80 | 9.80 | 9.80 | 9.80 |
| Methionine | 3.40 | 3.50 | 3.50 | 3.50 |

^1^The mineral premix provided the following mineral elements for per kg of diet: Fe (as ferrous sulfate) 218.82 mg, Cu (as copper sulfate) 16 mg, Zn (as zinc sulfate) 173.91 mg, Mn (as manganese sulfate) 6.29 mg, I (as potassium iodide) 3.68 mg and Se (as sodium selenite) 20 mg.

^2^The vitamin premix provided the following vitamins for per kg of diet: Vitamin A 5512 IU, Vitamin D_3_ (25-hydroxy) 2250 IU, Vitamin E 24 mg, Vitamin K_3_ 3 mg, Vitamin B_1_ 3 mg, Vitamin B_2_ 6 mg, Vitamin B_6_ 3 mg, Vitamin B_12_ 24 μg, pantothenic acid 15 mg, folic acid 1.2 mg and biotin 150 μg.

^3^The contents of DE, CP, EE, CF, NDF and ADF were measured values, while the contents of DF, calcium, AP, lysine and methionine were calculated values.

^4^The content of DF was calculated according to the following formula: DF = SDF + IDF.

CON, control group; PF, 15% pea-hull fiber in the diet; OB: 15% oat bran in the diet; MIX, 7.5% pea-hull fiber and 7.5% oat bran in the diet.

**Supplemental Table 2.** The content of main nutrients in raw pea-hull fiber and oat bran used in the current study^1^

| Nutrient content, % | Pea-hull fiber | | Oat bran |
| --- | --- | --- | --- |
| Dry matter | 78.4 | | 81.1 |
| Crude protein | 6.69 | | 17.6 |
| Ether extract | 0.650 | | 8.90 |
| Crude fiber | 44.2 | | 5.61 |
| NDF | 61.7 | | 17.9 |
| ADF | 50.9 | | 11.6 |
| Crude ash | 3.05 | | 5.27 |
| SDF | 4.67 | 12.8 | |
| IDF | 74.5 | 35.7 | |

NDF, Neutral detergent fibers. ADF, Acid detergent fiber. SDF, Soluble dietary fiber. IDF, Insoluble dietary fiber.

**Supplemental Table 3.** The sequences of primers using in current study

| Gene^1^ | Primer sequence (5’-3’) | Product length (bp) | Accession no. |
| --- | --- | --- | --- |
| *β-actin* | F:TCTGGCACC AC ACCTTCT  R:TGATCTGGGTCATCTTCTCAC | 132 | DQ178122 |
| *GAPDH* | F:TTTGCGTCAGTGTCATCG  R:TGCTCTGCCTTGGGTAAT | 220 | NM_001206359.1 |
| *18s rRNA* | F:CTGCCTTCCTTGGATGTG  R:GCGGCTTTGGTGACTCTA | 195 | AY265350 |
| *ZO-1* | F：TGGCATTATTCGCCTTCATAC  R：AGCCTCATTCGCATTGTTT | 171 | AJ318101.1 |
| *ZO-2* | F：ATCGATATGGAGGAGGTGATATGG  R：CTCGAGACTTTTCTATTAACTTCCGAGC | 163 | NM_011597 |
| *OCLN* | F：CTACTCGTCCAACGGGAAAG  R：ACGCCTCCAAGTTACCACTG | 158 | NM_001163647.1 |
| *MUC1* | F: GTGCCGCTGCCCACAACCTG  R: AGCCGGGTACCCCAGACCCA | 190 | XM_001926883.4 |
| *MUC2* | F: CTGCTCTTGGGCACTATATG  R: CCTGTGACTGCAGAATCAAC | 170 | XM_003122394.1 |
| *pBD-1* | F: TGCCACAGGTGCCGATCT  R: CTGTTAGCTGCTTAAGGAATAAAGGC | 81 | 396819 |
| *NOD1* | F：CTGTCGTCAACACCGATCCA  R:CCAGTTGGTGACGCAGCTT | 57 | AB187219.1 |
| *NOD2* | F:GAGCGCATCCTCTTAACTTTCG  R： ACGCTCGTGATCCGTGAAC | 55 | AB195466.1 |
| *TLR4* | F：CAGATAAGCGAGGCCGTCATT  R：TTGCAGCCCACAAAAAGCA | 113 | AB232527 |
| *MyD88* | F：GATGGTAGCGGTTGTCTCTGAT  R：GATGCTGGGGAACTCTTTCTTC | 148 | AB292176.1 |
| *IRAK4* | F: CAAGGCAGGTCAGGTTTCGT  R: TTCGTGGGGCGTGTAGTGT | 115 | XM_003135490.1 |
| *TRAF6* | F: CAAGAGAATACCCAGTCGCACA  R: ATCCGAGACAAAGGGGAAGAA | 267 | NM_001105286.1 |
| *PIPK2* | F: CAGTGTCCAGTAAATCGCAGTTG  R: CAGGCTTCCGTCATCTGGTT | 206 | XM_003355027.1 |
| *IL-1β* | F: GCATGTGCTGAGCCTTTGTA  R:CCTGGTCCTCCCAAGATTGT | 181 | NM_214055.1 |
| *IL-6* | F:GGCTGCTTCTGGTGATGC  R:AGAGATTTTGCCGAGGATGTA | 146 | M80258 |
| *IL-10* | F: GACCAGTGGGCGACTTCTT  R: ACGGCCTTGCTCTTGTTTTC | 242 | L20001 |

^1^GAPDH, glyceraldehyde 3 phosphate dehydrogenase; *ZO-1*, zonula occludens 1; *ZO-2*, zonula occludens 2; *OCLN*, occluding; *MUC1*, mucin1; *MUC2*, mucin2; *pBD-1*, porcine β defensins-1; *NOD1*, nucleotide binding oligomerization domain protein receptor 1; *NOD2*, nucleotide binding oligomerization domain protein receptor 2; *TLR4*, toll like receptor 4; *MyD88*, myeloid differentiation factor 88; *IRAK4*, interleukin-1 receptor associated kinase; *TRAF6*, tumor necrosis factor receptor associated factor 6; *PIPK2*, receptor-interacting serine /threonine protein kinase 2; *IL-1β*, interleukin 1β; *IL-6*, interleukin 6; *IL-10*, interleukin 10.

**Supplemental Table 4** The components of SDS-PAGE^1^

| Ingredients | Separation gel (mL) | Spacer gel (mL) |
| --- | --- | --- |
| Double distilled water | 1.025 | 1.4 |
| Acrylamide/N,N'-methylene bisacrylamide (30%) | 1.025 | 0.33 |
| 1.0 M Tris-HCl) (*pH*=8.8) | 2.0 | / |
| 1.0 M Tris-HCl (*pH*=6.8) | / | 0.25 |
| 10% SDS | 0.05 | 0.02 |
| 10% APS^3^ | 0.05 | 0.02 |
| TEMED^4^ | 0.002 | 0.002 |
| Total volume | 5 | 2 |

^1^SDS-PAGE, polyacrylamide gel electrophoresis

^2^Tris-HCl, trihydroxymethylaminomethane-HCl

^3^APS, ammonium persulfate

^4^TEMED: Tetramethylethylenediamine

**Supplemental Table 5** The growth performance of pigs in CON, PF, OB and MIX groups^1^

| Item | CON | PF | OB | MIX | SEM | *P* value |
| --- | --- | --- | --- | --- | --- | --- |
| Initial weight (kg) | 32.3 | 32.50 | 32.4 | 32.4 | 0.38 | 1.00 |
| Final weight (kg) | 81.4^a^ | 72.9^b^ | 77.8^a^ | 78.4^a^ | 1.26 | 0.02 |
| FI (kg/d) | 2.21^a^ | 1.94^b^ | 1.99^a^ | 2.02^a^ | 0.04 | 0.02 |
| BWG (g/d) | 876^a^ | 690^b^ | 810^a^ | 821^a^ | 222 | 0.01 |

^1^The variant alphabetical superscript in the same row indicates significant difference when *P* < 0.05, n = 6. CON, control group; PF, 15% pea-hull fiber in the diet; OB, 15% oat bran in the diet; MIX, 7.5% pea-hull fiber and 7.5% oat bran in the diet. FI, feed intake; BWG, body weight gain. SEM, standard error of mean.

**Supplemental Table 6** The relative abundance of bacterial phyla in the ileal and colonic digesta from pigs in the four different groups (%)^1^

| Phylum | Ileum | | | | | | Colon | | | | | |
| --- | --- | --- | --- | --- | --- | --- | --- | --- | --- | --- | --- | --- |
|  | CON | PF | OB | MIX | IQR^2^ | *P* value | CON | PF | OB | MIX | IQR^2^ | *P* value |
| Actinobacteria | 0.105 | 0.066 | 0.0656 | 0.0518 | 0.01 | 0.57 | 0.396 | 0.109 | 0.0838 | 0.0899 | 0.10 | 0.63 |
| Bacteroidetes | 0.468 | 0.490 | 0.143 | 0.835 | 0.13 | 0.26 | 47.8 | 40.7 | 42.7 | 43.6 | 1.60 | 0.44 |
| Cyanobacteria | 0.0857 | 0.116 | 0.296 | 0.205 | 0.04 | 0.34 | 0.0324 | 0.015 | 0.0699 | 0.0576 | 0.06 | 0.46 |
| Deferribacteres | - | - | - | - | - | - | 0.0028 | 0.0012 | 0.0094 | 0.0528 | 0.01 | 0.82 |
| Fibrobacteres | - | - | - | - | - | - | 0.0561 | 0.0358 | 0.0587 | 0.182 | 0.06 | 0.62 |
| Firmicutes | 75.9 | 83.5 | 91.3 | 84.4 | 23.49 | 0.49 | 41.0 | 42.8 | 44.9 | 46.8 | 1.30 | 0.44 |
| Lentisphaerae | - | - | - | - | - | - | 0.0802 | 0.0349 | 0.121 | 0.0711 | 0.08 | 0.20 |
| Planctomycetes | - | - | - | - | - | - | 0.0827 | 0.0257 | 0.062 | 0.0761 | 0.09 | 0.40 |
| Proteobacteria | 23.2 | 15.5 | 7.96 | 14.1 | 22.51 | 0.46 | 4.67 | 7.87 | 3.74 | 4.03 | 0.70 | 0.14 |
| Spirochaetes | 0.0282 | 0.0412 | 0.0204 | 0.0670 | 0.07 | 0.57 | 3.09 | 4.09 | 5.07 | 3.61 | 0.83 | 0.87 |
| Synergistetes | - | - | - | - | - | - | 0.012 | 0.0026 | 0.0101 | 0.0169 | 0.01 | 0.24 |
| Tenericutes | 0.0193 | 0.0362 | 0.0078 | 0.154 | 0.02 | 0.54 | 0.236 | 0.251 | 0.316 | 0.283 | 0.27 | 0.35 |
| WPS-2 | - | - | - | - | - | - | 0.0043^b^ | 0.0024^b^ | 0.0473^a^ | 0.0056^b^ | 0.02 | <0.01 |

^1^The variant alphabetical superscript in the same row indicates significant difference when *P* < 0.05, n = 6. CON, control group; PF, 15% pea-hull fiber in the diet; OB: 15% oat bran in the diet; MIX, 7.5% pea-hull fiber and 7.5% oat bran in the diet. The symbol ‘-‘ means undetected.

^2^IQR, interquartile range. For those normally distributed data, IQR is replaced by the standard error of mean. Of all data, the relative abundance of Actinobacteria, Bacteroidetes and Cyanobacteria in the ileal digesta of the pigs, and the abundance of Bacteroidetes, Firmicutes, Proteobacteria and Spirochaetes in the colonic digesta of the pigs is normally distributed.

**Supplemental Table 7**. The relative abundance of top 20 identified OTUs at genus level in the ileal and colonic digesta from pigs in CON, PF, OB and MIX groups (%)1

| Genus | Ileum | | | | | | | Colon | | | | | | | |
| --- | --- | --- | --- | --- | --- | --- | --- | --- | --- | --- | --- | --- | --- | --- | --- |
|  | Phylum | CON | PF | OB | MIX | IQR^2^ | *P* value | Genus | Phylum | CON | PF | OB | MIX | SEM^3^ | *P* value |
| *Actinobacillus* | Proteobacteria | 5.34 | 0.570 | 0.947 | 0.770 | 4.58 | 0.58 | *Anaerovibrio* | Firmicutes | 1.04 | 1.40 | 0.69 | 0.55 | 0.17 | 0.32 |
| *Citrobacter* | Proteobacteria | 0.131 | 0.391 | 0.0578 | 0.177 | 0.29 | 0.18 | *Bacteroidales*;f_;g_ | Bacteroidetes | 3.94 | 2.81 | 2.26 | 2.15 | 1.98 | 0.43 |
| *Clostridiaceae*;g_ | Firmicutes | 32.7^ab^ | 3.43^b^ | 61.9^a^ | 23.7^b^ | 31.87 | < 0.01 | CF231 | Bacteroidetes | 1.67^a^ | 0.859^b^ | 1.20^ab^ | 0.872^b^ | 0.11 | 0.02 |
| *Clostridiaceae*;Other | Firmicutes | 0.934^a^ | 0.164^b^ | 0.583^ab^ | 0.160^b^ | 0.46 | 0.02 | *Clostridiaceae*;g_ | Firmicutes | 1.57^ab^ | 1.16^b^ | 2.54^a^ | 1.24^ab^ | 1.68 | 0.06 |
| *Clostridiales*;f_;g_ | Firmicutes | 8.42 | 0.441 | 1.90 | 1.04 | 3.13 | 0.07 | *Clostridiales*;f_;g_ | Firmicutes | 5.95 | 6.47 | 7.73 | 6.81 | 0.41 | 0.50 |
| *Clostridiales*;Other;Other | Firmicutes | 0.292^ab^ | 0.132^b^ | 0.502^a^ | 0.198 ^ab^ | 0.38 | 0.03 | *Coprococcus* | Firmicutes | 1.48 | 1.9 | 0.93 | 1.55 | 0.21 | 0.46 |
| *Clostridium* | Firmicutes | 1.47 | 0.264 | 0.747 | 0.418^ab^ | 0.66 | 0.09 | *Enterobacteriaceae*; g_ | Proteobacteria | 0.391 | 2.78 | 1.12 | 1.40 | 0.52 | 0.45 |
| *Enterobacteriaceae*;g_ | Proteobacteria | 11.7 | 3.65 | 1.49 | 2.39 | 5.93 | 0.41 | *Lachnospira* | Firmicutes | 0.627^b^ | 1.08^ab^ | 0.413^b^ | 2.72^a^ | 0.28 | <0.01 |
| *Enterobacteriaceae*;Other | Proteobacteria | 0.808 | 2.27 | 0.368 | 0.963 | 0.31 | 0.14 | *Lachnospiraceae*;g_ | Firmicutes | 4.54 | 4.02 | 5.89 | 5.3 | 3.11 | 0.45 |
| *Lactobacillales*;f_;g_ | Firmicutes | 0.178 | 0.257 | 0.0961 | 0.323 | 0.06 | 0.50 | *Lactobacillus* | Firmicutes | 1.33 | 3.41 | 1.54 | 4.25 | 0.51 | 0.11 |
| *Lactobacillus* | Firmicutes | 1.86 | 80.8 | 0.969 | 4.54 | 62.03 | 0.08 | *Megasphaera* | Firmicutes | 1.26 | 2.07 | 1.24 | 3.25 | 0.44 | 0.34 |
| *Peptostreptococcaceae*;g_ | Firmicutes | 0.988 | 0.171 | 1.88 | 0.262 | 0.57 | 0.23 | *Oscillospira* | Firmicutes | 1.89 | 2.06 | 2.51 | 1.83 | 0.19 | 0.59 |
| *Prevotella* | Bacteroidetes | 0.188 | 0.177 | 0.0593 | 0.335 | 0.05 | 0.26 | *Phascolarctobacterium* | Firmicutes | 0.71 | 0.866 | 0.459 | 0.752 | 0.06 | 0.10 |
| *Pseudomonadaceae*;g_ | Proteobacteria | 0.515 | 0.917 | 0.262 | 1.34 | 0.95 | 0.14 | *Prevotella* | Bacteroidetes | 32.8 | 29.1 | 29.6 | 31.3 | 2.24 | 0.95 |
| *Pseudomonas* | Proteobacteria | 0.0819 | 0.307 | 0.125 | 0.117 | 0.27 | 0.37 | *Ruminococcaceae*;g_ | Firmicutes | 8.09 | 8.09 | 9.02 | 7.36 | 0.61 | 0.83 |
| *Ruminococcaceae*;g_ | Firmicutes | 0.116 | 0.0773 | 0.0605 | 0.530 | 0.11 | 0.57 | *Ruminococcus* | Firmicutes | 1.24 | 2.13 | 1.51 | 2.00 | 0.24 | 0.55 |
| SMB53 | Firmicutes | 0.246 | 0.0492 | 0.169 | 0.0337 | 0.12 | 0.05 | S24-7;g_ | Bacteroidetes | 6.72 | 5.53 | 7.49 | 8.03 | 0.7 | 0.64 |
| *Streptococcus* | Firmicutes | 1.71 | 0.361 | 5.48 | 0.239 | 6.42 | 0.50 | *Streptococcus* | Firmicutes | 3.96^a^ | 0.68^b^ | 2.45^a^ | 1.01^b^ | 1.86 | 0.04 |
| *Streptophyta*;g_ | Cyanobacteria | 0.0845 | 0.116 | 0.296 | 0.203 | 0.04 | 0.34 | *Succinivibrio* | Proteobacteria | 1.91 | 3.07 | 0.730 | 1.79 | 0.51 | 0.47 |
| *Turicibacter* | Firmicutes | 18.2^a^ | 4.05^b^ | 11.4^ab^ | 5.26^ab^ | 1.87 | 0.03 | *Treponema* | Spirochaetes | 3.09 | 4.08 | 5.08 | 3.62 | 3.45 | 0.57 |

^1^The variant alphabetical superscript in the same row indicates significant difference between groups when *P* < 0.05, n = 6. CON, control group; PF, 15% pea-hull fiber in the diet; OB: 15% oat bran in the diet; MIX, 7.5% pea-hull fiber and 7.5% oat bran in the diet. f_, unidentified family; g_, unidentified genus; Other, unknown family or genus.

^2^IQR, interquartile range. For those normally distributed data, IQR is replaced with SEM (standard error of mean). Of all genera shown in this table, the relative abundance of *Enterobacteriaceae*;Other, *Lactobacillales*;f_;g_, *Prevotella*, *Streptophyta*;g_ and *Prevotella* is normally distributed.

^3^SEM is replaced with IQR when the data is non-normally distributed. Of all genera shown in this table, the relative abundance of *Bacteroidales*;f_;g_, *Clostridiaceae*;g_, *Lachnospiraceae*;g_, *Streptococcus* and *Succinivibrio* is non- normally distributed.


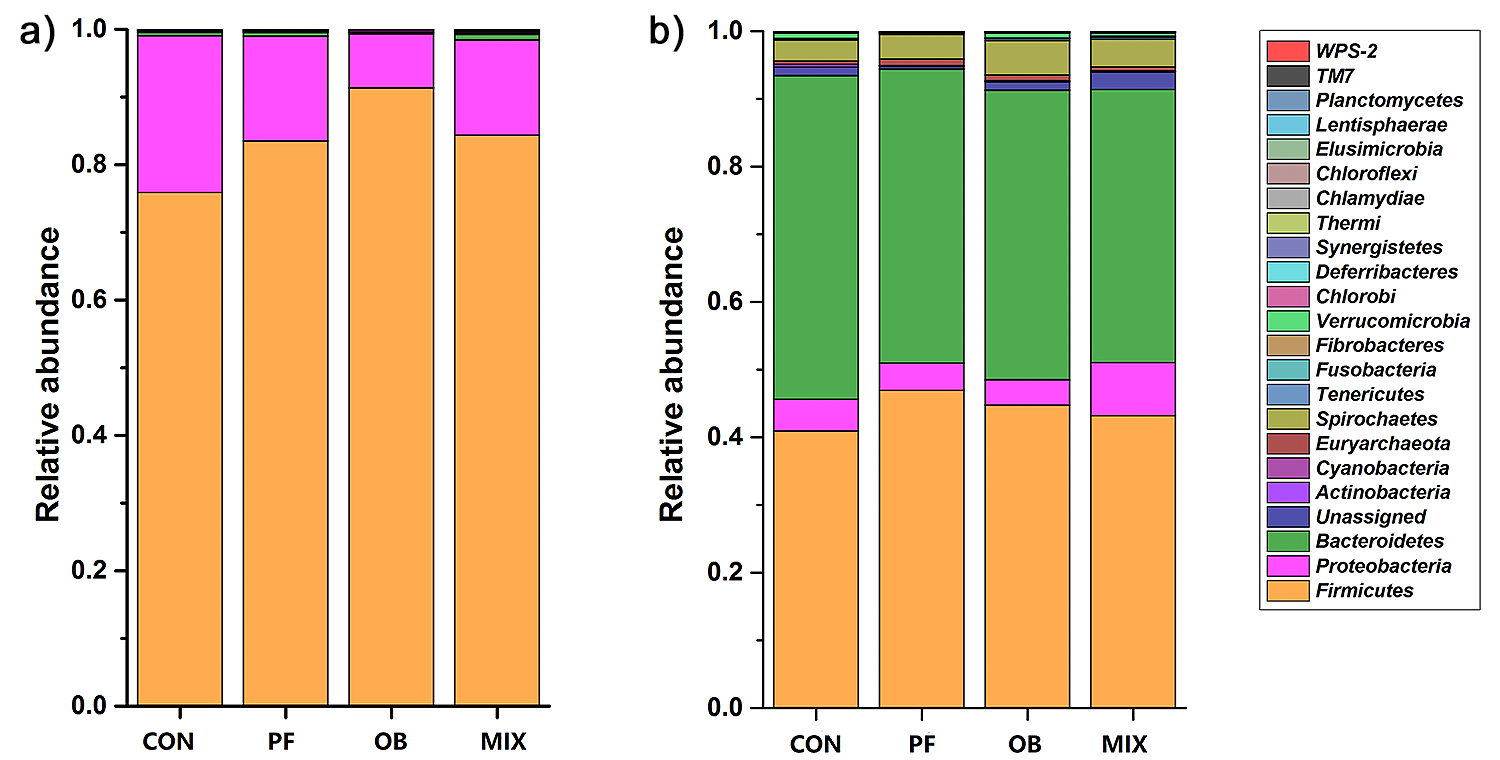


**Supplemental Figure 1**. The composition of microbes in the ileal and colonic digesta samples from pigs in the four different groups (phylum). CON, control group; PF, 15% pea-hull fiber in the diet; OB: 15% oat bran in the diet; MIX, 7.5% pea-hull fiber and 7.5% oat bran in the diet. n = 6.


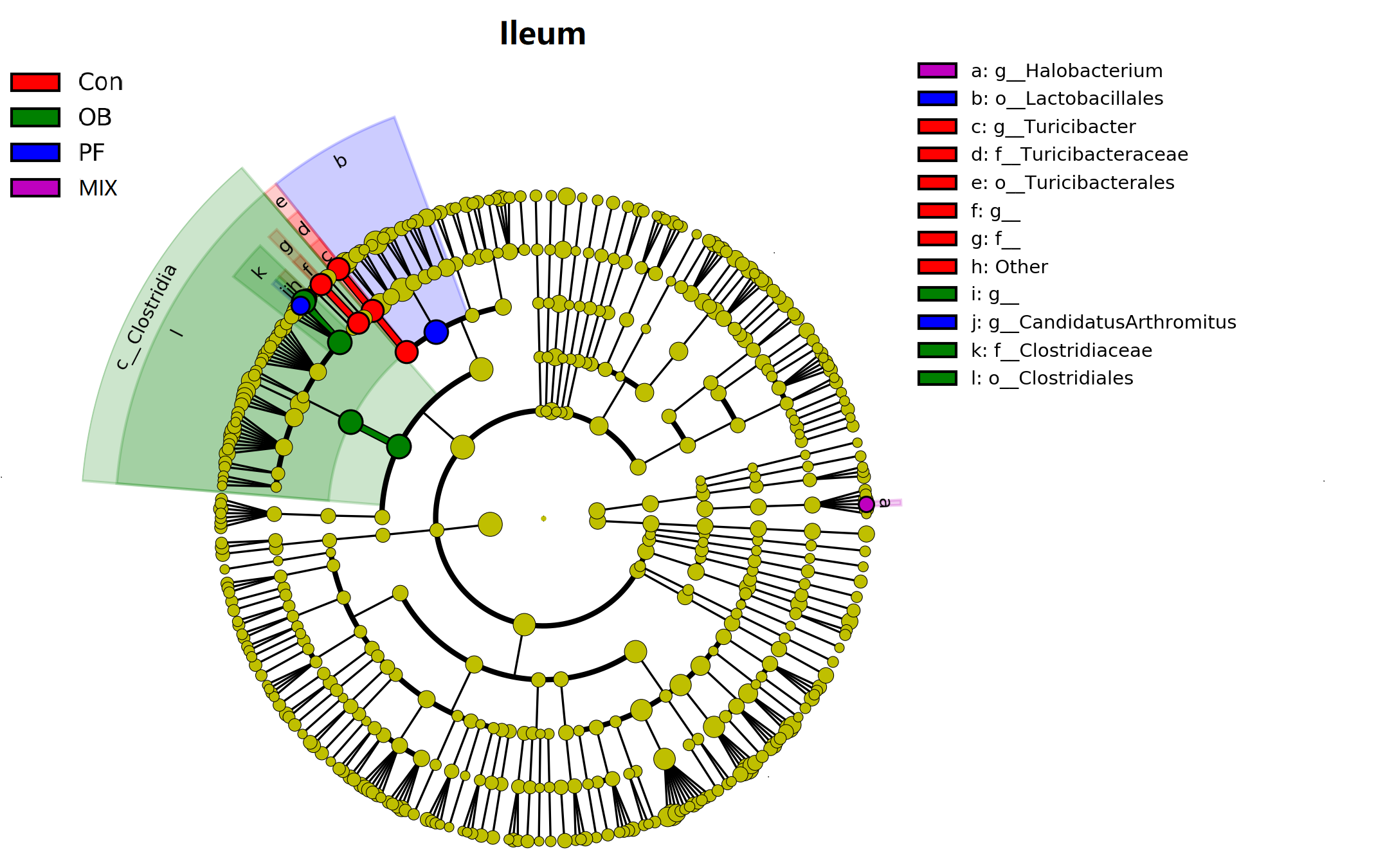


**Supplemental Figure 2** Histogram of the linear discriminant analysis (LDA) scores computed for bacterial taxa differentially abundant in the ileum of pigs among the four groups using default parameters (α = 0.01, LDA score > 3.0, n = 6). Six levels of the biomarkers (from phylum to genus) were shown in this figure. The circles radiating from inner to outside mean levels from phylum to genus. Small spots on each circle mean sub-classifications on the corresponding level, and the size of each spot is in proportion to the corresponding sub-classification. Sub-classifications with no differences among the treatments are colored with yellow, while biomarkers in each treatment are colored with different colors. A total of 12 differentially abundant bacterial taxa were detected. Of those, only one taxon was significantly overrepresented in the ileum of pigs fed mixed fibers containing diet (purple), two taxa were overrepresented in the ileum of pigs fed pea-hull fiber containing diet (blue), three taxa were overrepresented in the ileum of pigs fed oat bran containing diet (green), and six taxa were overrepresented in the ileum of controlled pigs (red). CON, control group; PF, 15% pea-hull fiber in the diet; OB: 15% oat bran in the diet; MIX, 7.5% pea-hull fiber and 7.5% oat bran in the diet.


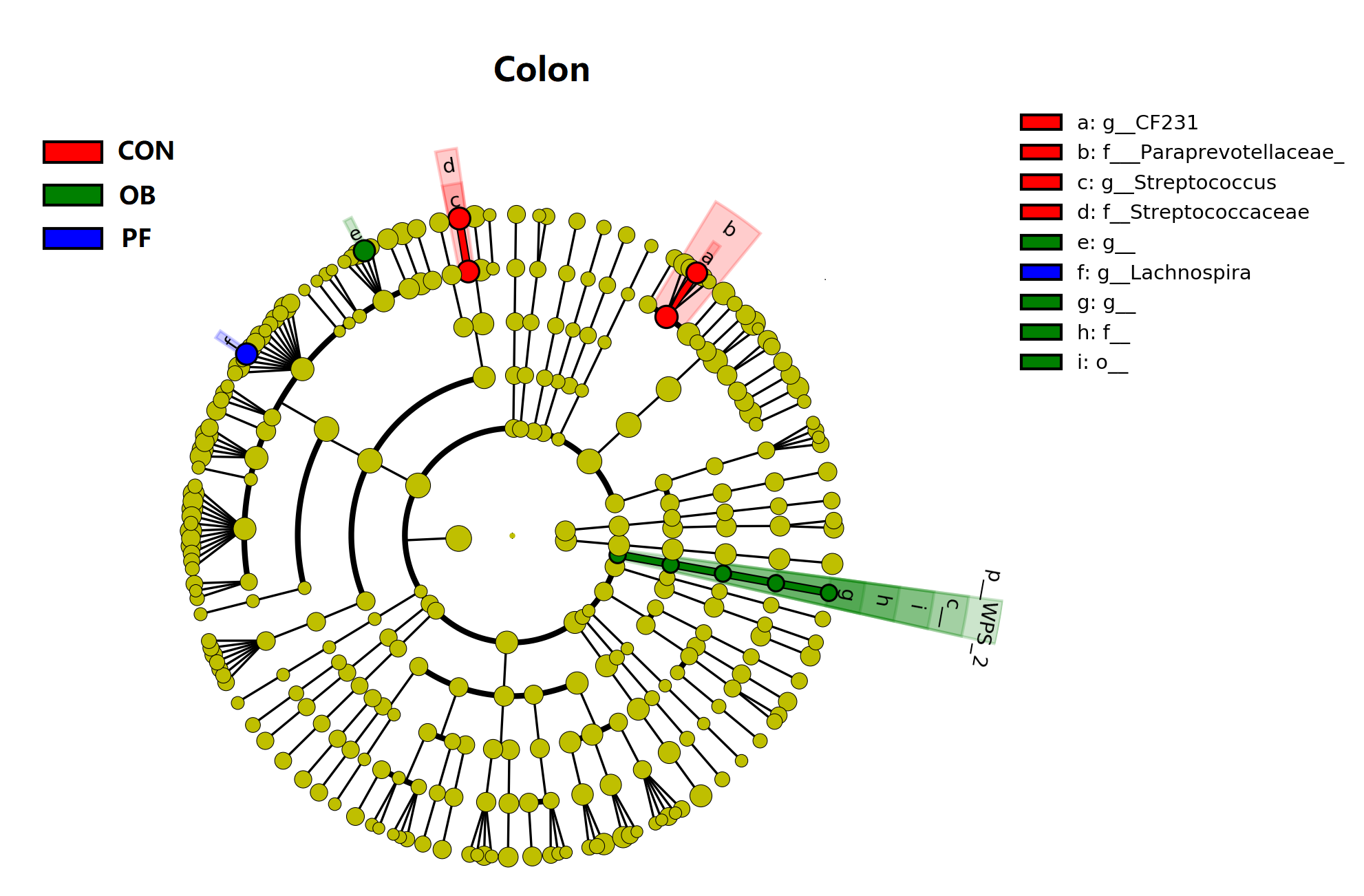


**Supplemental Figure 3** Histogram of the linear discriminant analysis (LDA) scores computed for bacterial taxa differentially abundant in the colon of pigs among the four groups using default parameters (α = 0.01, LDA score > 3.0, n = 6). A total of 9 differentially abundant bacterial taxa were detected. Of those, only one taxon was significantly overrepresented in the colon of pigs fed pea-hull fiber containing diet (blue), four taxa were overrepresented in the colon of pigs fed oat bran containing diet (green), and four taxa were overrepresented in the colon of controlled pigs (red). CON, control group; PF, 15% pea-hull fiber in the diet; OB: 15% oat bran in the diet; MIX, 7.5% pea-hull fiber and 7.5% oat bran in the diet.
